# Pesticide Use and Relative Leukocyte Telomere Length in the Agricultural Health Study

**DOI:** 10.1371/journal.pone.0133382

**Published:** 2015-07-21

**Authors:** Gabriella Andreotti, Jane A. Hoppin, Lifang Hou, Stella Koutros, Shahinaz M. Gadalla, Sharon A. Savage, Jay Lubin, Aaron Blair, Mirjam Hoxha, Andrea Baccarelli, Dale Sandler, Michael Alavanja, Laura E. Beane Freeman

**Affiliations:** 1 Division of Cancer Epidemiology and Genetics, National Cancer Institute, National Institutes of Health, Department of Health and Human Services, Bethesda, Maryland, United States of America; 2 Department of Biological Sciences, North Carolina State University, Raleigh, North Carolina, United States of America; 3 Department of Preventive Medicine and Robert H. Lurie Comprehensive Cancer Center, Feinberg School of Medicine, Northwestern University, Chicago, Illinois, United States of America; 4 Department of Clinical Sciences and Community Health, Center of Molecular and Genetic Epidemiology, University of Milan, Milan, Italy; 5 Laboratory of Environmental Epigenetics, Exposure Epidemiology and Risk Program, Department of Environmental Health, Harvard School of Public Health, Boston, Massachusetts, United States of America; 6 Epidemiology Branch, National Institute of Environmental Health Sciences, NIH/DHHS, Research Triangle Park, North Carolina, United States of America; Hasselt University, BELGIUM

## Abstract

Some studies suggest that telomere length (TL) may be influenced by environmental exposures, including pesticides. We examined associations between occupational pesticide use reported at three time points and relative telomere length (RTL) in the Agricultural Health Study (AHS), a prospective cohort study of pesticide applicators in Iowa and North Carolina. RTL was measured by qPCR using leukocyte DNA from 568 cancer-free male AHS participants aged 31-94 years with blood samples collected between 2006 and 2008. Self-reported information, including pesticide use, was collected at three time points: enrollment (1993-1997) and two follow-up questionnaires (1998-2003, 2005-2008). For each pesticide, we evaluated cumulative use (using data from all three questionnaires), and more recent use (using data from the last follow-up questionnaire). Multivariable linear regression was used to examine the associations between pesticide use (ever, lifetime days, intensity-weighted lifetime days (lifetime days*intensity score)) and RTL, adjusting for age at blood draw and use of other pesticides. Of the 57 pesticides evaluated with cumulative use, increasing lifetime days of 2,4-D (p-trend=0.001), diazinon (p-trend=0.002), and butylate (p-trend=0.01) were significantly associated with shorter RTL, while increasing lifetime days of alachlor was significantly associated with longer RTL (p-trend=0.03). Only the association with 2,4-D was significant after adjustment for multiple comparisons. Of the 40 pesticides evaluated for recent use, malathion was associated with shorter RTL (p=0.03), and alachlor with longer RTL (p=0.03). Our findings suggest that leukocyte TL may be impacted by cumulative use and recent use of certain pesticides.

## Introduction

Telomeres are long, repetitive nucleotide sequences located at chromosome ends that are essential for genomic integrity [1]. Telomere length (TL) typically shortens with age due to incomplete replication of telomeric DNA with each cell division. When telomeres reach a critically short length, cellular apoptosis or senescence is triggered causing cell death [1].

There is increasing evidence that environmental and occupational exposures may be linked to either shorter or longer changes in TL. For example, a recent review of 14 epidemiologic studies identified 11 studies that reported shorter TL with exposure to various exposures including, pesticides, polycyclic aromatic hydrocarbons, benzene, carbon black, n-nitrosamine, landfill waste, particulate matter, and lead [2]. Ten of these 11 studies examined blood cell TL, and one examined buccal cell TL. The remaining three studies found longer blood cell TL with exposure to arsenic, persistent organic pollutants (including organochlorine pesticides and polychlorinated biphenyls), and short-term exposure to particulate matter [2]. Little is known about the effects of pesticides on TL. Two of the 14 studies in the review paper examined pesticide use, which were case-control studies of myelodsyplastic syndrome (MDS) and found shorter blood cell TL among those occupationally exposed to any, unspecified pesticides [3,4]. To our knowledge, only one study has examined the relationship between specific pesticide use and relative telomere length (RTL); this study was conducted in the same cohort we are using, the Agricultural Health Study (AHS), and found that lifetime use of 2,4-D, alachlor, metolachlor, trifluarlin, DDT, permethrin, and toxaphene were linked to shorter buccal cell RTL compared to those who did not use of each pesticide [5].

Further examination of the relationship between pesticides and RTL is important because it may provide insight into the mechanism linking pesticides with the development and/or progression of some diseases. Furthermore, little is known about the timing of environmental exposures (e.g., cumulative versus recent exposure) on TL, or if environmental exposures impact TL differently by cell type (e.g., buccal versus blood TL). In this study, we examined cumulative and more recent pesticide use with RTL measured in blood DNA from participants in the AHS.

## Methods

### Study Population

A description of the AHS population has been previously published [6,7]. Briefly, it is a prospective cohort study of licensed private and commercial pesticide applicators, and spouses of private applicators, residing in Iowa and North Carolina. Applicators were enrolled between 1993 and 1997 when they applied for pesticide licenses. At enrollment, participants completed a self-administered questionnaire and were given another questionnaire to complete at home. Spouses of enrolled applicators were asked to complete a questionnaire and return it by mail or complete it over the phone. Subsequently, two follow-up phone interviews were conducted approximately 5 years apart (1998–2003 and 2005–2008). Participants provided written consent when they completed the enrollment questionnaire. The relevant institutional review boards (National Institutes of Health, Westat, and University of Iowa) approved the AHS and participant consent procedures, as well as this study.

Participants in this analysis were male private applicators who provided a blood sample between 2006 and 2008 as part of a sub-study focused on neurobehavioral outcomes [7]. Of the 612 participants in the neurobehavioral sub-study with sufficient DNA, TL analysis was complete for 568 cancer-free participants.

### Pesticide Exposure

At enrollment, participants provided information on their lifetime use of 50 specific pesticides, while the two follow-up questionnaires allowed participants to provide information on any pesticide they used since the previous questionnaire. Three metrics of pesticide use (ever use, lifetime days (days per year x number of years), and intensity-weighted lifetime days (lifetime days x intensity score)) were developed [8]. Briefly, the intensity score takes into account the application methods and personal protective equipment commonly reported by study participants. For this analysis we categorized lifetime and intensity-weighted lifetime days into tertiles (low, medium, high).

To obtain cumulative use for each individual pesticide, we combined data from the questionnaires for 83 pesticides, utilizing similar methods at described by Hoppin *et al* [9]. Of these pesticides, we had data from all three questionnaires for 50 pesticides, and data from the two follow-up questionnaires for the remaining 33 pesticides. Thus, exposure data for the 50 pesticides includes use prior to enrollment through the second follow-up questionnaire, while exposure data for the 33 pesticides includes use after enrollment though the second follow-up questionnaire. We excluded 26 pesticides that were used by less than 5% of the study population, leaving 57 pesticides for analysis of cumulative use.

We also examined more recent pesticide use; this was restricted to use reported on the second follow-up questionnaire (2005–2008), which collected information on pesticide use since the completion of the first follow-up questionnaire. Thus, the more recent use analysis captured pesticide use between 1999 and 2008. We do not have the date of last pesticide use, but know that the time between completion of the second follow-up questionnaire and blood draw ranged from 0.4 to 2.2 years, with a mean of 1.2 years. For the more recent pesticide use analysis, we further excluded 17 pesticides that were used by fewer than 5 people, leaving 40 pesticides for analysis.

### Relative Telomere Length (RTL) Measurement

DNA was extracted from whole blood using DNAQuik reagents and stored at -80°C after extraction. RTL analysis was conducted at the Laboratory of Environmental Epigenetics, Center of Molecular and Genetic Epidemiology, Department of Environmental and Occupational Health of Milan University, Italy using quantitative real-time PCR as described previously [5,10]. Briefly, this method determines RTL in genomic DNA by determining the ratio of telomere repeat copy number (T) to single copy gene (S) (human β-globin gene) copy number (T/S ratio) in individual samples relative to a reference pooled DNA [11]. The reference pooled DNA was created using samples from 60 participants randomly selected from the population sample for this study, and used to generate a standard curve, ranging from 0.25 to 8 ng/μl in every T and S PCR run. All samples were successfully run in triplicate with a 100% completion rate. For quality control purposes, we included 12 duplicate samples; the inter-batch variability, or coefficient of variation (CV) was 6.2%.

### Statistical Analysis

The mean of the triplicate RTL measurements were used in statistical analyses. RTL had a right-skewed distribution and a statistically significant Kolmogorov-Smirnov test for normality (p<0.01); therefore, we used the natural logarithm of RTL for statistical analyses. We computed the mean RTL and standard errors (SE) for selected characteristics reported on the last follow-up questionnaire (race, state of residence, education, cigarette smoking status, drank alcohol last year, and BMI) using a general linear model adjusting for age at blood draw. The same statistical method was used to compute age-adjusted mean RTL for the three metrics of pesticide use. Linear regression was used to test for trend associations between tertiles of each pesticide (including no use) and RTL, adjusting for age at blood draw. We accounted for potential confounding from use of other pesticides by further adjusting for the most highly correlated pesticide that also showed an association with RTL. Other potential confounders, including state of residence, smoking, BMI, and total lifetime days of pesticide use, were evaluated but not retained in the final models since they did not affect results measurably. For the recent pesticide use analysis, we accounted for prior use of the same pesticide by including covariates for use reported at enrollment and the first follow-up questionnaire in the statistical model. To take into account the multiple associations tested, we calculated the False Discovery Rate (FDR) using the p-trend values for lifetime days of each pesticide [12]. All statistical analyses were conducted using SAS 9.2 (SAS, Cary, N.C.)

### Buccal—blood comparison

Of the 568 study participants, 40 were also in the previous AHS analysis using buccal cell samples [5]. Briefly, this previous study included 1,234 AHS participants who provided a mouthwash sample between 1999 and 2006 (between 5–8 years prior to blood collection), and evaluated lifetime pesticide use reported at enrollment. DNA was extracted using the Wizard Genomics DNA Purification Lit (Promega Corp., Madison, WI, USA), and RTL was measured in the same laboratory and by the same method as this study, but approximately 2 years earlier. We calculated mean buccal and blood RTL for selected characteristics using the statistical methods described above, and examined the correlations between buccal and blood RTL using Spearman correlation coefficient.

## Results

Among the 568 participants, the mean RTL and standard error (SE) was 1.09 (0.02), with a range from 0.30 to 6.64. All participants were male, 98% self-reported as white, and the mean age at blood draw was 59 years, with a range from 31 to 94 years (Table 1). As expected, RTL decreased with increasing continuous age at blood draw (p-trend = 0.01; Spearman correlation r = -0.16, p = 0.001). By design, half of the study population was from NC and half from IA, 43% were ever cigarette smokers and 63% consumed alcohol in the past year. For BMI, 50% were overweight and 32% obese. Other than age, none of the demographic factors examined, including cigarette packs per years and smokeless tobacco use were statistically significantly associated with RTL.

**Table 1:** Mean relative telomere length by selected characteristics.

Selected Characteristics^1^	N	%	Mean RTL^2^	SE^2^	P^2^ ^,^ ^3^
**Total**	**568**	**100.0**	**1.09**	**0.02**	-
**Age at blood draw**					
31–51	147	25.9	1.12	0.05	
52–58	136	23.9	1.20	0.05	
59–68	154	27.1	1.05	0.04	
69–94	131	23.1	0.97	0.05	0.01
**Race**					
White	541	97.8	1.09	0.02	
Black	8	1.4	1.09	0.20	
Other	4	0.7	0.79	0.28	0.56
**State of Residence**					
North Carolina	283	49.8	1.12	0.03	
Iowa	285	50.2	1.06	0.03	0.21
**Education**					
High school or less	274	49.5	1.09	0.03	
More than high school	279	50.5	1.08	0.03	0.89
**Cigarette smoking**					
Never	325	57.4	1.06	0.03	
Former	184	32.5	1.03	0.13	
Current	40	7.1	1.15	0.09	
Not regular	17	3.0	1.08	0.09	0.31
**Drank alcohol last year**					
Never	212	37.4	1.13	0.04	
Ever	355	62.6	1.12	0.03	0.16
**Body Mass Index (kg/m** ^**2**^ **)**					
<18.5	1	0.2	-	-	
18.5–24	103	18.2	1.05	0.06	
25–29	283	50.0	1.08	0.03	
≥30	179	31.6	1.12	0.04	0.12

^1^Selected characterisitics ascertained at last follow-up, except age at blood draw

^2^Age at blood draw

^3^F-test for difference in mean using natural log of TL

### Cumulative Pesticide Use

Of the 57 pesticides examined for cumulative use, 15 showed some evidence of association with age-adjusted RTL (P<0.10) for at least one of the three metrics of exposure (data not shown). For each of these 15 pesticides, we further adjusted for the individual pesticide that was most highly correlated with the pesticide of interest and was linked to RTL (S1 Table). The Spearman correlations between correlated pesticides ranged between 0.20–0.54. Adjusting for age and correlated pesticide use, cumulative use of four pesticides was significantly associated with RTL (Table 2). For ever *vs*. never use, we found significant associations for 2,4-D (p = 0.01) and butylate (p = 0.04) with shorter RTL, and a borderline significant association for aldrin and shorter RTL (p = 0.05). Significant exposure-response associations were seen for tertiles of lifetime days of 2,4-D (p-trend = 0.001), diazinon (p-trend = 0.002) and butylate (p-trend = 0.01) with shorter RTL. We also saw a significant association for alachlor and longer RTL (p-trend = 0.03). The associations for lifetime intensity-weighted days were similar to lifetime days. The FDR accounting for the 57 comparisons, resulted in borderline significant associations for cumulative use of 2,4-D (p FDR = 0.05) and diazinon (p FDR = 0.06) (data not shown). Associations between cumulative pesticide use and RTL were not measurably changed when we further adjusted for state of residence, smoking, BMI, and total lifetime days of pesticide use.

**Table 2:** Cumulative lifetime pesticide use and mean relative telomere length.

	Ever Use				Lifetime Days^1^				Lifetime Intensity Weighted Days^2^			
Cumulative Pesticide Use^3^	N	Mean RTL^4^	SE^4^	P^4^ ^,^ ^5^	N	Mean RTL^4^	SE^4^	P^4^ ^,^ ^5^	N	Mean RTL^4^	SE^4^	P^4^ ^,^ ^5^
**HERBICIDE**												
**Alachlor** ^**a**^												
Not Used	187	1.04	0.04		187	1.04	0.04		187	1.04	0.04	
Used	381	1.11	0.03	0.17	-	-	-		-	-	-	
Low	-	-	-		135	1.05	0.05		121	1.06	0.05	
Medium	-	-	-		109	1.17	0.05		122	1.15	0.05	
High	-	-	-		124	1.17	0.05	0.03	122	1.18	0.05	0.04
**Butylate** ^**a**^												
Not Used	344	1.10	0.03		344	1.10	0.03		344	1.10	0.03	
Used	224	1.02	0.04	0.04	-	-	-		-	-	-	
Low	-	-	-	-	55	0.96	0.07		54	0.98	0.07	
Medium	-	-	-	-	48	1.05	0.07		56	1.01	0.07	
High	-	-	-	-	63	0.98	0.06	0.01	55	1.00	0.07	0.01
**2,4-D** ^**b**^												
Not Used	99	1.22	0.06		99	1.22	0.06		99	1.22	0.06	
Used	469	1.03	0.03	0.01	-	-	-		-	-	-	
Low	-	-	-	-	155	1.17	0.05		151	1.11	0.05	
Medium	-	-	-	-	145	1.04	0.05		152	1.10	0.05	
High	-	-	-	-	168	1.01	0.04	0.001	152	0.96	0.05	0.0003
**INSECTICIDE**												
**Diazinon** ^**c**^												
Not Used	284	1.15	0.04		284	1.15	0.04		284	1.15	0.04	
Used	284	1.06	0.03	0.15	-	-	-		-	-	-	
Low	-	-	-	-	73	1.11	0.07		62	1.13	0.07	
Medium	-	-	-	-	55	0.99	0.07		63	1.03	0.07	
High	-	-	-	-	64	0.98	0.07	0.002	63	0.99	0.07	0.004

^1^ Days per year x number of years

^2^ Lifetime days x intensity score

^3^ Cumulative use from enrollment and two follow-up questionnaires

^4^ Adjusted for age at blood draw and: ^a^metolachlor, ^b^alachlor, ^c^metalaxyl

^5^ Linear regression using log(RTL) and categorical pesticide use (never, low, medium, high)

### Recent Pesticide Use

Of the 40 pesticides examined for more recent pesticide use, seven showed some evidence of association with RTL adjusting for age and prior use of the same pesticide (p<0.10) (data not shown). After further adjusting for the most highly correlated pesticide that was also linked to RTL, we found significant associations between recent use of alachlor and longer RTL (p = 0.03) and between recent use of malathion and shorter RTL (p = 0.03) (Table 3). Accounting for the 40 comparisons, neither of these associations was statistically significant at an FDR of ≤0.05. Associations between recent pesticide use and RTL were not measurably changed when we further adjusted for state of residence, smoking, BMI, and total lifetime days of pesticide use.

**Table 3:** Recent pesticide use and mean relative telomere length.

Recent Pesticide Use^1^	N	Mean RTL^2^	SE^2^	P^2^ ^,^ ^3^
**HERBICIDE**				
**Alachlor** ^**a**^				
Not Used	531	1.06	0.03	
Used	37	1.33	0.09	0.03
**INSECTICIDE**				
**Malathion** ^**b**^				
Not Used	473	1.03	0.05	
Used	95	0.92	0.07	0.03

^1^ Recent pesticide use reported at second follow-up questionnaire (2005–2008)

^2^ Adjusted for age at blood draw and recent use of: ^a^metolachlor, ^b^diazinon

^3^ Linear regression using log(TL) and adjusting for use at enrollment and first follow-up questionnaire

### Buccal—Blood Comparison

For the 40 participants in this sub-analysis, the age at buccal cell collection ranged from 48–89 years, while age at blood collection ranged from 55–94 years of age. There was a mean of 6.3 years between buccal and blood collection (range: 5–8 years). The distributions of race, state of residence, education, cigarette smoking, alcohol drinking, and BMI were consistent with those reported for the respective parent study populations. Among the 40 participants, age-adjusted mean RTL was 1.00 in the blood sample and 1.14 in the buccal sample. There was little correlation between age-adjusted mean RTL in buccal and blood (r = -0.14, p = 0.34). Given the limitations of this sub-analysis, including the small sample size, difference in time periods of collection and analysis, and lack of correlation between buccal and blood RTL, the associations between pesticides with buccal and blood RTL could not be meaningfully compared.

## Discussion

In this study of male pesticide applicators, we found significant exposure-response relationships for cumulative use of 2,4-D, diazinon and butylate with shorter RTL, as well as for cumulative use of alachlor and longer RTL. After taking multiple comparisons into account, the strongest exposure-response association was for cumulative use of 2,4-D and shorter RTL. Recent use of alachlor and malathionwere linked to shorter and longer RTL, respectively.

2,4-D (2,4-Dichlorophenoxyacetic acid) is a chlorophenoxy herbicide that is commonly used to control broadleaf weeds [13] its cumulative prevalence in the study population is 83%. The association between higher 2,4-D use and shorter RTL was also found in the previous AHS analysis looking at lifetime pesticide use reported at enrollment and buccal RTL [5]. Little is known about the molecular effects of 2,4-D in exposed humans. A small study of twelve 2,4-D applicators and nine controls found increased lymphocyte replicative index among the applicators versus the controls, as well as a higher replicative index among applicators after spraying than before spraying [14]. Consistent with these findings, a subsequent study found an in-vivo increase in replicative index at a low dose of commercial 2,4-D [15]. Replicative index is an indicator of cell proliferation, which is a key factor in carcinogenesis [14, 15]. Agricultural 2,4-D use has been linked to higher incidence of cancer, predominately Non-Hodgkin lymphoma (NHL) [16]; and urinary 2,4-D levels were linked with markers of myocardial infarction and type-2 diabetes in a NHANES II study [17]. Together these findings suggest that TL may be an intermediate marker connecting 2,4-D use and disease risk.

Recent use of two pesticides, alachlor, a chloroacetanilide herbicide, and malathion, an organophosphate insecticide, were significantly associated with RTL. Both cumulative and more recent use of alachlor were linked with RTL, while only recent use of malathion was associated with RTL, suggesting an acute or temporary effect of this pesticide. No previous studies have examined temporality of pesticide use on RTL, and very little is known in general about the effects of exposure duration or timing on changes in TL. Two studies of particulate matter in occupational populations found that recent exposure was associated with changes in TL [18,19]. Dioni *et al* found significantly longer TL 3 days after exposure [18]; while the study by Wong *et a*l found that exposure a month prior resulted in significantly shorter TL [19]. Our results, together with these previous findings suggest that acute exposures may be linked to longer or shorter TL.

Overall, our findings add to the growing evidence linking environmental/occupational exposures with changes in RTL. Consistent with our findings, most prior epidemiologic studies of environmental/occupational exposures have reported associations with shorter TL [2]. However, we did find one pesticide, alachlor, was linked with longer RTL. This may reflect a chemical-specific mechanism or may be due to chance, since we did not find significant associations for other chloroacetanilide herbicides. Associations with longer TL have been reported, including a study among healthy Koreans that found a significant correlation between organochlorine pesticides, particularly DDE, and longer TL [20]. In terms of biological mechanisms, it has been suggested that environmental/occupational exposures, including pesticides, can increase the presence of reactive oxidative species, leading to oxidative stress and DNA damage [21,22]. Studies have reported that oxidative stress causes DNA damage to telomeric regions due to their guanine-rich sequences and lack of protective proteins, which can result in telomere shortening [23,24,25]. In contrast, experimental studies have shown telomere lengthening in response to increased telomerase activity during acute inflammation [26]. Several other proteins can affect TL, which is a complex process that is not fully understood [1,15,26]. Both shorter and TL have linked to various diseases; most studies to date have reported associations between shorter TL and higher cancer risk [27,28], although longer TL has also been implicated [29].

In our sub-analysis of 40 participants we found no correlation between blood and buccal RTL. Given this lack of correlation and the small sample size, we did not evaluate pesticide use with RTL in this sub-group. To our knowledge, no previous studies have examined the effect of environmental or occupational exposures on TL by cell type in the same population. Because pesticides are absorbed and metabolized differently by different organs and tissues [30], it is plausible that pesticides impact buccal and blood cells differently. Studies comparing intra-individual TL by cell type are limited, but have shown differences. For example, in a study of bone marrow failure patients, TL was longest in fibroblasts and shortest in blood; but intra-individual TL shortening between cell types were significantly correlated (blood–buccal cells r = 0.74) [31]. Similarly, in another study of skeletal muscle, skin and subcutaneous fat, TL differed between tissue types, but telomere shortening was significantly correlated (r = 0.72–0.84) [32]. To more accurately assess the impact of pesticide use on different cell types, the samples should be collected at the same time, and laboratory methods should be identical.

Comparing our findings in blood lymphocytes to that of Hou *et al*, where TL was measured in buccal cells, only the association for cumulative exposure to 2,4-D and shortened telomeres was consistent. No other associations were consistent in both studies. Furthermore the association for increasing cumulative alachlor use and TL was in the opposite direction, with shorter telomeres observed in the previous analysis, but longer telomeres in this analysis. This could be due to differences between the two studies, including cell type, time of sample collection, sub-populations, and exposure time periods. Chance findings due to multiple comparisons are also a possibility.

In this study, we were able to examine several specific pesticides in a prospective cohort of pesticide applicators with longitudinal data and reliable histories of pesticide use [33,34]. We were also able to examine the exposure-response relationships using data from three questionnaires spanning over 10 years, as well as more recent pesticide use. However, because we did not know the exact time between last pesticide use and blood collection, our interpretation of the findings are limited. Data on demographic characteristics were obtained from the last follow-up questionnaire, which was administered on average 1.2 years prior to blood collection. Although the sample size was small, we did examine the correlation of TL in blood and buccal samples. Since little is known about the correlations between TL in different media, and the effects of pesticides on the human body, our findings are an important contribution warranting further research. In our analysis, we were able to assess the impact of a number of confounding factors and control for the use of other pesticides. However, as with any cross-sectional measurement, we were unable to adjust for TL at baseline. Although we considered multiple comparisons in our analysis, we cannot rule out chance findings.

In conclusion, these findings suggest that cumulative and more recent use of certain pesticides may be linked to alterations in RTL, which may be a potential intermediate in certain diseases. The strongest association was for 2,4-D and shorter TL, which was borderline significant after accounting for multiple comparisons and was consistent with Hou *et al*. Future studies examining associations between pesticide use, TL and disease outcome are needed to more thoroughly examine this hypothesis.

## Supporting Information

S1 Table Cumulative lifetime pesticide use and mean relative telomere length. (DOC)
