# Supplementary material for: Pesticide Use and Relative Leukocyte Telomere Length in the Agricultural Health Study
Source: PLoS One. 2015 Jul 21;10(7):e0133382. doi: 10.1371/journal.pone.0133382 (PMC4510132; doi:10.1371/journal.pone.0133382)
Supplement: S1 Table — (DOC) [file pone.0133382.s001.doc]

| **Supplemental Table 1. Cumulative lifetime pesticide use and mean relative telomere length** | | | | | | | | | | | | |  |  |  |
| --- | --- | --- | --- | --- | --- | --- | --- | --- | --- | --- | --- | --- | --- | --- | --- |
|  |  |  |  |  |  |  |  |  |  |  |  |  |  |  |  |
|  | **Ever Use** | | | |  | **Lifetime Days^1^** | | | |  | **Lifetime Intensity  Weighted Days^2^** | | | |  |
| **Cumulative Pesticide Use^3^** | **N** | **Mean RTL^4^** | **SE^4^** | **P^4,5^** |  | **N** | **Mean RTL^4^** | **SE^4^** | **P^4,5^** |  | **N** | **Mean RTL^4^** | **SE^4^** | **P^4,5^** |  |
| **HERBICIDE** |  |  |  |  |  |  |  |  |  |  |  |  |  |  |  |
| **Alachlor^a^** |  |  |  |  |  |  |  |  |  |  |  |  |  |  |  |
| Not Used | 187 | 1.04 | 0.04 |  |  | 187 | 1.04 | 0.04 |  |  | 187 | 1.04 | 0.04 |  |  |
| Used | 381 | 1.11 | 0.03 | 0.17 |  | - | - | - |  |  | - | - | - |  |  |
| Low | - | - | - |  |  | 135 | 1.05 | 0.05 |  |  | 121 | 1.06 | 0.05 |  |  |
| Medium | - | - | - |  |  | 109 | 1.17 | 0.05 |  |  | 122 | 1.15 | 0.05 |  |  |
| High | - | - | - |  |  | 124 | 1.17 | 0.05 | 0.03 |  | 122 | 1.18 | 0.05 | 0.04 |  |
| **Butylate^a^** |  |  |  |  |  |  |  |  |  |  |  |  |  |  |  |
| Not Used | 344 | 1.10 | 0.03 |  |  | 344 | 1.10 | 0.03 |  |  | 344 | 1.10 | 0.03 |  |  |
| Used | 224 | 1.02 | 0.04 | 0.04 |  | - | - | - |  |  | - | - | - |  |  |
| Low | - | - | - | - |  | 55 | 0.96 | 0.07 |  |  | 54 | 0.98 | 0.07 |  |  |
| Medium | - | - | - | - |  | 48 | 1.05 | 0.07 |  |  | 56 | 1.01 | 0.07 |  |  |
| High | - | - | - | - |  | 63 | 0.98 | 0.06 | 0.01 |  | 55 | 1.00 | 0.07 | 0.01 |  |
| **Metolachlor^b^** |  |  |  |  |  |  |  |  |  |  |  |  |  |  |  |
| Not Used | 267 | 1.04 | 0.03 |  |  | 267 | 1.04 | 0.03 |  |  | 267 | 1.04 | 0.03 |  |  |
| Used | 301 | 1.11 | 0.04 | 0.19 |  | - | - | - |  |  | - | - | - |  |  |
| Low | - | - | - |  |  | 98 | 1.12 | 0.06 |  |  | 98 | 1.19 | 0.06 |  |  |
| Medium | - | - | - |  |  | 100 | 1.09 | 0.06 |  |  | 95 | 1.09 | 0.06 |  |  |
| High | - | - | - |  |  | 99 | 1.17 | 0.06 | 0.29 |  | 95 | 1.08 | 0.06 | 0.99 |  |
| **Paraquat^c^** |  |  |  |  |  |  |  |  |  |  |  |  |  |  |  |
| Not Used | 361 | 1.12 | 0.04 |  |  | 361 | 1.12 | 0.04 |  |  | 361 | 1.12 | 0.04 |  |  |
| Used | 207 | 1.10 | 0.04 | 0.88 |  | - | - | - |  |  | - | - | - |  |  |
| Low | - | - | - | - |  | 68 | 1.06 | 0.07 |  |  | 45 | 0.99 | 0.09 |  |  |
| Medium | - | - | - | - |  | 29 | 1.20 | 0.11 |  |  | 49 | 1.20 | 0.08 |  |  |
| High | - | - | - | - |  | 49 | 1.19 | 0.08 | 0.51 |  | 47 | 1.27 | 0.09 | 0.13 |  |
| **Sethoxydim^d^** |  |  |  |  |  |  |  |  |  |  |  |  |  |  |  |
| Not Used | 530 | 1.10 | 0.03 |  |  | 530 | 1.10 | 0.03 |  |  | 530 | 1.10 | 0.03 |  |  |
| Used | 38 | 1.23 | 0.09 | 0.18 |  | - | - | - |  |  | - | - | - |  |  |
| Low | - | - | - | - |  | 10 | 1.30 | 0.21 |  |  | 7 | 1.33 | 0.24 |  |  |
| Medium | - | - | - | - |  | 15 | 1.07 | 0.18 |  |  | 9 | 0.85 | 0.24 |  |  |
| High | - | - | - | - |  | 13 | 1.52 | 0.19 | 0.10 |  | 8 | 1.37 | 0.20 | 0.10 |  |
| **2,4-D^b^** |  |  |  |  |  |  |  |  |  |  |  |  |  |  |  |
| Not Used | 99 | 1.22 | 0.06 |  |  | 99 | 1.22 | 0.06 |  |  | 99 | 1.22 | 0.06 |  |  |
| Used | 469 | 1.03 | 0.03 | 0.01 |  | - | - | - |  |  | - | - | - |  |  |
| Low | - | - | - | - |  | 155 | 1.17 | 0.05 |  |  | 151 | 1.11 | 0.05 |  |  |
| Medium | - | - | - | - |  | 145 | 1.04 | 0.05 |  |  | 152 | 1.10 | 0.05 |  |  |
| High | - | - | - | - |  | 168 | 1.01 | 0.04 | 0.001 |  | 152 | 0.96 | 0.05 | 0.0003 |  |
| **2,4,5-TP^e^** |  |  |  |  |  |  |  |  |  |  |  |  |  |  |  |
| Not Used | 484 | 1.08 | 0.03 |  |  | 484 | 1.08 | 0.03 |  |  | 484 | 1.08 | 0.03 |  |  |
| Used | 84 | 1.25 | 0.06 | 0.13 |  | - | - | - |  |  | - | - | - |  |  |
| Low | - | - | - | - |  | 11 | 0.91 | 0.14 |  |  | 11 | 0.94 | 0.14 |  |  |
| Medium | - | - | - | - |  | 10 | 1.20 | 0.14 |  |  | 13 | 1.10 | 0.12 |  |  |
| High | - | - | - | - |  | 15 | 1.13 | 0.12 | 0.75 |  | 12 | 1.27 | 0.13 | 0.29 |  |
| **INSECTICIDE** |  |  |  |  |  |  |  |  |  |  |  |  |  |  |  |
| **Aldrin^f^** |  |  |  |  |  |  |  |  |  |  |  |  |  |  |  |
| Not Used | 429 | 1.13 | 0.04 |  |  | 429 | 1.13 | 0.04 |  |  | 429 | 1.13 | 0.04 |  |  |
| Used | 139 | 1.04 | 0.05 | 0.05 |  | - | - | - |  |  | - | - | - |  |  |
| Low | - | - | - | - |  | 12 | 0.90 | 0.16 |  |  | 28 | 0.97 | 0.11 |  |  |
| Medium | - | - | - | - |  | 34 | 0.96 | 0.10 |  |  | 32 | 0.88 | 0.09 |  |  |
| High | - | - | - | - |  | 44 | 0.89 | 0.09 | 0.07 |  | 30 | 0.92 | 0.11 | 0.06 |  |
| **DDT^g^** |  |  |  |  |  |  |  |  |  |  |  |  |  |  |  |
| Not Used | 355 | 0.99 | 0.04 |  |  | 355 | 0.99 | 0.04 |  |  | 355 | 0.99 | 0.04 |  |  |
| Used | 213 | 1.17 | 0.04 | 0.003 |  | - | - | - |  |  | - | - | - |  |  |
| Low | - | - | - | - |  | 61 | 1.04 | 0.07 |  |  | 48 | 1.08 | 0.08 |  |  |
| Medium | - | - | - | - |  | 13 | 1.02 | 0.16 |  |  | 49 | 0.98 | 0.08 |  |  |
| High | - | - | - | - |  | 72 | 1.08 | 0.07 | 0.10 |  | 49 | 1.10 | 0.08 | 0.10 |  |
| **Diazinon^h^** |  |  |  |  |  |  |  |  |  |  |  |  |  |  |  |
| Not Used | 284 | 1.15 | 0.04 |  |  | 284 | 1.15 | 0.04 |  |  | 284 | 1.15 | 0.04 |  |  |
| Used | 284 | 1.06 | 0.03 | 0.15 |  | - | - | - |  |  | - | - | - |  |  |
| Low | - | - | - | - |  | 73 | 1.11 | 0.07 |  |  | 62 | 1.13 | 0.07 |  |  |
| Medium | - | - | - | - |  | 55 | 0.99 | 0.07 |  |  | 63 | 1.03 | 0.07 |  |  |
| High | - | - | - | - |  | 64 | 0.98 | 0.07 | 0.002 |  | 63 | 0.99 | 0.07 | 0.004 |  |
| **Heptachlor^i^** |  |  |  |  |  |  |  |  |  |  |  |  |  |  |  |
| Not Used | 454 | 1.05 | 0.03 |  |  | 454 | 1.05 | 0.03 |  |  | 454 | 1.05 | 0.03 |  |  |
| Used | 114 | 1.11 | 0.05 | 0.79 |  | - | - | - |  |  | - | - | - |  |  |
| Low | - | - | - | - |  | 11 | 0.93 | 0.16 |  |  | 23 | 0.91 | 0.11 |  |  |
| Medium | - | - | - | - |  | 23 | 0.91 | 0.11 |  |  | 20 | 0.92 | 0.13 |  |  |
| High | - | - | - | - |  | 33 | 0.94 | 0.10 | 0.29 |  | 24 | 0.96 | 0.11 | 0.40 |  |
| **FUNGICIDE** |  |  |  |  |  |  |  |  |  |  |  |  |  |  |  |
| **Benomyl^d^** |  |  |  |  |  |  |  |  |  |  |  |  |  |  |  |
| Not Used | 431 | 1.13 | 0.05 |  |  | 431 | 1.13 | 0.05 |  |  | 431 | 1.13 | 0.05 |  |  |
| Used | 137 | 1.15 | 0.05 | 0.58 |  | - | - | - |  |  | - | - | - |  |  |
| Low | - | - | - |  |  | 26 | 1.02 | 0.11 |  |  | 30 | 1.09 | 0.10 |  |  |
| Medium | - | - | - |  |  | 37 | 1.14 | 0.10 |  |  | 31 | 1.10 | 0.10 |  |  |
| High | - | - | - |  |  | 29 | 1.11 | 0.11 | 0.50 |  | 31 | 1.09 | 0.11 | 0.65 |  |
| **Chlorothalonil^c^** |  |  |  |  |  |  |  |  |  |  |  |  |  |  |  |
| Not Used | 464 | 1.10 | 0.03 |  |  | 464 | 1.10 | 0.03 |  |  | 464 | 1.10 | 0.03 |  |  |
| Used | 104 | 1.14 | 0.06 | 0.25 |  | - | - | - |  |  | - | - | - |  |  |
| Low | - | - | - |  |  | 28 | 1.10 | 0.11 |  |  | 31 | 1.10 | 0.11 |  |  |
| Medium | - | - | - |  |  | 37 | 1.19 | 0.09 |  |  | 33 | 1.17 | 0.09 |  |  |
| High | - | - | - |  |  | 33 | 1.15 | 0.10 | 0.38 |  | 32 | 1.23 | 0.11 | 0.17 |  |
| **Maneb/Mancozeb^c^** | |  |  |  |  |  |  |  |  |  |  |  |  |  |  |
| Not Used | 477 | 1.08 | 0.03 |  |  | 477 | 1.08 | 0.03 |  |  | 477 | 1.08 | 0.03 |  |  |
| Used | 91 | 1.19 | 0.06 | 0.31 |  | - | - | - |  |  | - | - | - |  |  |
| Low | - | - | - | - |  | 15 | 0.98 | 0.15 |  |  | 21 | 1.01 | 0.13 |  |  |
| Medium | - | - | - | - |  | 28 | 1.10 | 0.10 |  |  | 21 | 1.06 | 0.12 |  |  |
| High | - | - | - | - |  | 21 | 1.25 | 0.12 | 0.62 |  | 22 | 1.28 | 0.12 | 0.51 |  |
| **Metalaxyl^c^** |  |  |  |  |  |  |  |  |  |  |  |  |  |  |  |
| Not Used | 374 | 1.09 | 0.04 |  |  | 374 | 1.09 | 0.04 |  |  | 374 | 1.09 | 0.04 |  |  |
| Used | 194 | 1.13 | 0.04 | 0.75 |  | - | - | - |  |  | - | - | - |  |  |
| Low | - | - | - | - |  | 47 | 1.11 | 0.08 |  |  | 52 | 1.07 | 0.08 |  |  |
| Medium | - | - | - | - |  | 49 | 1.15 | 0.08 |  |  | 55 | 1.18 | 0.08 |  |  |
| High | - | - | - | - |  | 67 | 1.19 | 0.07 | 0.23 |  | 54 | 1.23 | 0.08 | 0.15 |  |
| ^1^ Days per year x number of years | | |  |  |  |  |  |  |  |  |  |  |  |  |  |
| ^2^ Lifetime days x intensity score | | |  |  |  |  |  |  |  |  |  |  |  |  |  |
| ^3^ Cumulative use from enrollment and two follow-up questionnaires; | | | | | | | | | |  |  |  |  |  |  |
| Sethoxydim use from two follow-up questionnaires | | | | | | | |  |  |  |  |  |  |  |  |
| ^4^ Adjusted for age at blood draw and: ^a^metolachlor, ^b^alachlor, ^c^benomyl, ^d^chlorothanonil, ^e^DDT, | | | | | | | | | | | | | |  |  |
| ^f^hepatachlor, ^g^aldrin, ^h^metalaxyl, ^i^maneb/mancozeb | | | | | | | |  |  |  |  |  |  |  |  |
| ^5^ Linear regression using log(RTL) and categorical pesticide use (never, low, medium, high) | | | | | | | | | | | | |  |  |  |
